# The roles, mechanism, and mobilization strategy of endogenous neural stem cells in brain injury

**DOI:** 10.3389/fnagi.2022.924262

**Published:** 2022-08-17

**Authors:** Haijing Liu, Tao Wei, Qin Huang, Wei Liu, Yaopeng Yang, Yaju Jin, Danli Wu, Kai Yuan, Pengyue Zhang

**Affiliations:** ^1^Key Laboratory of Acupuncture and Massage for Treatment of Encephalopathy, College of Acupuncture, Tuina and Rehabilitation, Yunnan University of Traditional Chinese Medicine, Kunming, China; ^2^Library, Kunming Medical University, Kunming, China; ^3^School of Continuing Education, Yunnan University of Traditional Chinese Medicine, Kunming, China; ^4^Department of Teaching Affairs and Administration, Kunming Medical University, Kunming, China; ^5^School of Public Health, Kunming Medical University, Kunming, China; ^6^Department of Pulmonary and Critical Care Medicine, The Sixth Affiliated Hospital of Kunming Medical University, Yuxi, China

**Keywords:** brain injury, neuroregeneration, endogenous neural stem cell, therapeutic mechanism, mobilization strategy, therapeutic approaches

## Abstract

Brain injury poses a heavy disease burden in the world, resulting in chronic deficits. Therapies for brain injuries have been focused on pharmacologic, small molecule, endocrine and cell-based therapies. Endogenous neural stem cells (eNSCs) are a group of stem cells which can be activated *in vivo* by damage, neurotrophic factors, physical factor stimulation, and physical exercise. The activated eNSCs can proliferate, migrate and differentiate into neuron, oligodendrocyte and astrocyte, and play an important role in brain injury repair and neural plasticity. The roles of eNSCs in the repair of brain injury include but are not limited to ameliorating cognitive function, improving learning and memory function, and promoting functional gait behaviors. The activation and mobilization of eNSCs is important to the repair of injured brain. In this review we describe the current knowledge of the common character of brain injury, the roles and mechanism of eNSCs in brain injury. And then we discuss the current mobilization strategy of eNSCs following brain injury. We hope that a comprehensive awareness of the roles and mobilization strategy of eNSCs in the repair of cerebral ischemia may help to find some new therapeutic targets and strategy for treatment of stroke.

## Introduction

The brain is the center of the nervous system and controls all of our functions, including voluntary and involuntary movement, the secretion of hormones, study and memory formation, and so on. The damage and impairment of the brain directly brings about dysfunction in the body. The neural networks composed with neurons and their microenvironment is the functional foundation of the brain. And the destruction of neural networks is the common character of brain injury, such as stroke, trauma, cerebral palsy, neurodegenerative disorder, et al. (Larivière et al., 2019). Thus the reconstruction of the injured neural network is the ultimate purpose of the treatment for brain injury.

The key components in the reconstruction and repairmen of the neural network are neurogenesis and synaptogenesis (Ceanga et al., 2021; Puderbaugh and Emmady, 2022). The neurogenesis is the progress that new neurons generate and replace the injured neural cells in the adult brain after brain injury. And the synaptogenesis is the progress that neurites branch from the newborn neuron or injured neurons (with surviving bodies and dead axons or synapses), and repair the impaired neural networks. This progress needs a lot of newborn neurons derived from neural stem cells. Due to advances in brain biology, we already know that new neurons are generated from endogenous neural stem cells (eNSCs) throughout adulthood in the adult brain (Ottoboni et al., 2020). The eNSCs is a group of stem cells which have two essential properties: self-renewal and the potency of differentiation into neural cells. The most important neural stem cell pool in the adult brain is the subventricular zone (SVZ) and the dentate gyrus (DG) of hippocampus (Jinnou, 2021). These neural stem cells originated from SVZ and DG can proliferate, migrate and differentiate into neuron, oligodendrocyte and astrocyte. The newborn neurons can incorporate into network circuitry, the new oligodendrocytes can repair myelin sheaths, the new astrocytes can support, protect and nourish neural networks in the progress of reconstruction (Hamblin and Lee, 2021). Although the value and meaning of eNSCs in theory, the application of eNSCs in clinical setting limited by complex factors such as apoptosis, uncontrollable differentiation, and adverse microenvironment.

Thus a thorough clarification of the molecular mechanisms is essential for the application of eNSCs biology to promote repair strategies for brain injury. In this review, we firstly describe the current knowledge of the common character of brain injury, the roles and mechanism of eNSCs in brain injury. And then we discuss the current mobilization strategy of eNSCs following brain injury.

## The common character of brain injury: The destruction of neural networks

Brain injury refers to injuries to the cerebral hemispheres, cerebellum, and brain stem. According to the tree structure of brain injuries in Medical Subject Headings (MeSH) in National Library of Medicine (NLM), the major categories of brain injuries include brain trauma (traumatic brain hemorrhage, diffuse brain injuries, traumatic brain injuries, chronic brain injury, post-traumatic epilepsy), cerebral ischemia/hemorrhagic injury, degenerative injury.

### Ischemic stroke

Ischemic stroke caused by a disruption of blood flow to brain tissue leads to a lot of neuron loss and dysfunction, such as necrosis, apoptosis, autophagic cell death and synapse loss. The irreversible cerebral infarction is the primary lesion of ischemic stroke, which occurs within minutes in infarct core area, and gradually extends to the surrounding area of the lesion within a few hours (Feske, 2021). Ischemia leads to the malfunction of electron transport chain in the mitochondrial membrane, the calcium influx and calciumdependent excitotoxicity, the generation of reactive oxygen species, and ultimately leads to neural cells death. Meanwhile, ischemia triggers inflammatory response that leads to secondary brain injury and expand the infarction. These pathophysiology progress lead to the loss of the neuron and the synapse, and ultimately leads to the destruction of neural networks and neurological symptom (Ghelani et al., 2021; Wang et al., 2021). Neurological complications after ischemic stroke include brain edema, hemorrhagic transformation, seizures and epilepsy and delirium. Furthermore, deaths were one of the consequences of ischemic stroke within the first few days (Balami et al., 2011). During the acute phase of ischemic stroke, of all patients with the neurological worsening 33.6% of those encountered progressive stroke, 27.3% had brain swelling, 11.3% experienced a recurrent ischemic stroke, 10.5% suffered from parenchymal hemorrhage, and 17.3% had manifestations such as fever, hyperglycemia, and hypertension (Weimar et al., 2005).

### Cerebral hemorrhage

Non-traumatic intracerebral hemorrhage (ICH) caused by rupture of blood vessel in brain and extravasation of blood into the brain parenchyma. ICH is the subtype of stroke with the greatest mortality (Poon et al., 2015). According to the site of hemorrhage, the ICH divide into subarachnoid hemorrhage, subdural hemorrhage, lobar intracerebral hemorrhage and deep intracerebral hemorrhage (the basal ganglia, periventricular white matter, internal capsule, brainstem, and so on (Carpenter et al., 2016; Ziai and Carhuapoma, 2018). The hematoma and hydrocephalus cased by ICH not only destroy the neural cells in brain parenchyma and increases intracranial pressure, but also result in the ischemic brain injury in the areas far from the bleeding site, and exacerbate the brain injury (Bhattacharjee et al., 2021; Magid-Bernstein et al., 2022).

### Traumatic brain injury

Traumatic brain injury (TBI) is one of the most serious acute injuries with high incidence, high disability rate, and high mortality rate (Diaz-Arrastia, 2015). The TBI is usually caused by a violent bump, blow, or jolt to the head, or penetrating trauma, and the direct consequence of TBI is the damage of brain tissue and the destruction of neural networks induced by bruising, torn tissues, bleeding, neuroinflammation and apoptosis (Centers for Disease Control and Prevention (CDC), and National Center for Injury Prevention and Control., 2003; Peterson and Daugherty, 2019). In addition to acute injury, the chronic effects of TBI including impairment of motor, attention, memory, executive dysfunction and social deficits, can persist for months or years, and greatly reduce daily quality of life, and increase the burden for the patient and their family. The biological mechanisms involved in chronic TBI include neural apoptosis, neuroinflammatory reaction, demyelination, white matter pathology, which consistently damage the neural network and even lead to dementia (Fann et al., 2018; Haarbauer-Krupa et al., 2021).

### Neurodegenerative disease

Differing from the TBI and cerebral stroke, the neurodegenerative disease without significant loss of brain tissue is not caused by a sudden injury or cerebrovascular accident. The typical neurodegenerative diseases are Alzheimer’s and Parkinson’s disease. Alzheimer’s disease (AD) is a devastating neurodegenerative disorder that accounts for 70–80% of dementia (Reitz and Mayeux, 2011; Prince et al., 2013). The symptoms of AD include memory loss and cognitive decline. As far as we know now, the etiology of AD is the production, aggregation, and deposition of amyloid β (Aβ) peptides in the brain. The amyloidogenesis and deposition of Aβ generates excessive reactive oxygen species (ROS) and nitrogen species (RNS) which break the membranes and mitochondria of the neuron (Dobson, 2003; Rajasekhar and Govindaraju, 2015, 2018). Also, the Aβ initiates neuro-inflammation by activating glia and deteriorates the microenvironment of neural networks (Wyss-Coray, 2006). Ultimately, the neural networks are broken by oxidative stress, inflammation, and mitochondrial dysfunction. Parkinson’s disease (PD) is a severe movement disorder with muscle rigidity, tremors, and changes in speech and gait. The PD is caused by the dysfunction and death of neurons that produce dopamine in substantia nigra (SN) in the brain. Although the injury occurs in the local region in the brain, the related neural networks are badly affected by PD. The supplement and rescue of the neurons in SN can repair the impaired neural networks and help relieves symptoms (MedicinePlus, 2022).

Recently, frontotemporal lobe degeneration (FTD) has been attracted great attention. FTD present with a spectrum of syndromes characterized by neuronal degeneration in the frontal and anterior temporal lobes. FTD often leads to motor, language and memory disorders, it is the third most common cause of dementia. Some neurologists think that Parkinsonism is a manifestation of FTD, but some FTD patients are negative in the presynaptic dopaminergic deficiency when they are detected by dopamine transporter-single photon emission computed tomography (DaTSPECT). These finding implied that FTD is a neurodegenerative disease differentiated to the PD, although both of them lead to dementia (Menéndez-González et al., 2018). The underlying pathologic mechanism of FTD is intracellular deposition of abnormal aggregates of transactive DNA-binding protein TDP-43 in the frontal and temporal lobes, which lead to the degeneration of neurons and astrocytosis, and ultimately lead to frontotemporal lobar degeneration (Miki et al., 2020; Khan and De Jesus, 2022).

In summary, although the causes of these brain injuries are different in pathology and etiology, all of these brain injuries involves the loss of neurons and nerve tissue. This loss eventually leads to the dysfunction and destruction of neural networks. Thus the treatment of brain injury is not only to protect the neuron and its network in the early stage but also to repair the injured neural networks in the late stage.

## The roles and mechanism of endogenous neural stem cells in brain injury

Currently, the therapies for brain injuries have been focused on pharmacologic, rehabilitative training and cell-based therapies (Aertker and Cox, 2016; Szelenberger et al., 2020). Unfortunately, to date, we still lack effective pharmacologic in the treatment of brain injury because of the poor clinical effect and adverse side effects. Thus the cell-based therapies will become increasingly important in the treatment of brain injury. The cells used for neural repair can derive from activated eNSCs and cultured neural stem cells *in vitro* (Pereira and Rylander Ottosson, 2019). Numerous studies have confirmed that neural stem cell grafts promoted neural repair in brain injury. However, the great difficulty in cell source, transplantation methods, complex microenvironment and directed differentiation limited the application of stem cell transplantation. eNSCs have more advantages in the repair of the brain. Firstly, eNSCs do not initiate immune rejection and inflammatory response. Secondly, eNSCs are not affected by *in vitro* culture conditions and transplant operation, and the proliferation, generation and repair of eNSCs are sustainable. Thirdly, eNSCs will migrate to the site of injury under the action of chemokines. At last, eNSCs can migrate and differentiate in a more appropriate microenvironment. To date, extensive studies have shown that eNSCs have shown a beneficial effect in improving the functional recovery of the injured brain (Levison, 2018; Fang et al., 2019).

Factually, brain injury, such as stroke, trauma and neurodegeneration, activate the eNSCs (Liu et al., 1998; Wu, 1999; Gu and Wester, 2000; Nakagawa et al., 2000). Following stroke, in addition to the eNSCs derived from SVZ and DG, the local eNSCs in ischemic core and peri-infarct area, and microvascular pericytes residing near blood vessels can be activated and differentiate into neurosphere-like cell clusters expressing nestin (Nakagomi et al., 2009, 2011; Shimada et al., 2010; Takagi et al., 2017). In a photothrombotic stroke model, Vandeputte et al. (2014) found that 36% of eNSCs differentiate into astrocytes in the peri-infarct area and 21% into mature neurons at 90 days post stroke. In neonatal brain injury, the eNSCs derived from the ventricular-subventricular zone migrate to the damaged area and differentiate into neurons and oligodendrocytes, and improve functional gait behaviors (Felling et al., 2006; Jinnou, 2021). In an intraventricular hemorrhage model of rats, the proliferation and differentiation of eNSCs were enhanced by brain injury and the combination treatment with granulocyte colony stimulating factor (G-CSF) and lithium chloride, and attenuate the development of hydrocephalus and neuronal apoptosis (Yuan et al., 2016).

Similarly, TBI triggered the proliferation, differentiation of the eNSCs and migrated to the injured side (Sui and Jiang, 2016). The results from the group of Sun D showed that the proliferation of eNSCs in the DG had been activated by TBI in juvenile and adult rats. These eNSCs differentiated into mature neurons and integrated into the existing neuronal circuitry, and finally, promoted the cognitive recovery in the TBI model (Sun et al., 2005, 2007).

In a degenerative model of corticothalamic projection neurons in layer VI of the anterior cortex of mice, Magavi and Macklis (2000) and Macklis (2001) confirmed that the injury promoted neurogenesis and these newly generated eNSCs differentiated into mature neurons and formed long-distance corticothalamic connections. In animals with Huntington’s disease, the neurodegenerative process triggered immediately intensive cell proliferation and differentiation which led to the characteristic enlargement of the subependymal zone (SEZ) of lateral brain ventricles, which showed higher activation next to the degenerated striatum compared to the rostral part of the SEZ (R-SEZ). In the activated L-SEZ, niche cells with immature astrocytic phenotype ensheathed clusters of neural progenitors (Mazurová et al., 2006). Scharff et al. (2000) observed that the injured neurons caused by photolysis in the high vocal center (HVC) of adult songbirds were replaced and restore the impaired behavior. The neurogenesis was the potential therapeutic approaches of neurodegenerative disease including AD and PD (Lamm et al., 2014; Sung et al., 2020). Thus the restoration of neurogenesis and the replacement of injured neurons will be beneficial to the recovery of function from neurodegenerative disease (Yoneyama et al., 2011; van den Berge and Hol, 2013).

In summary, the injured brain retains the repair and regenerative potential to restore damaged neural networks through eNSCs (Table 1). And the mechanism of eNSCs involve a variety of mechanisms (Baker and West, 2019). The first is the neuronal replacement. Under an appropriate stimulus, the eNSCs primarily derived from SVA and DG can migrate to injured brain regions, differentiate into neurons, integrate into preexisting neural networks, and eventually replace the damaged neuron (Romanko et al., 2004). The second is remyelination. The eNSCs can differentiate into oligodendroglia cells and participate in myelination in the axons from newly generated neurons and the injured neurons (surviving bodies and dead axons), and play an important role in the reconstruction of the injured neural networks in brain injury (Kaneko and Sawamoto, 2013; Jinnou, 2021). The third is the secretion of trophic function. The neural stem cells can release neurotrophic factors such as nerve growth factor (NGF), brain derived neurotrophic factor (BDNF), and glial-derived neurotrophic factor (GDNF), and so on. These neurotrophic factors can improve the microenvironment of neural networks and promote nerve repair (Lu et al., 2003; Lladó et al., 2004). At last, the epigenetic mechanisms such as DNA methylation, histone acetylation and some microRNAs have been involved in the neurogenesis induced by brain injury (Liu et al., 2013, 2020).

**TABLE 1:** The roles, mechanism, and mobilization strategy of endogenous neural stem cells in brain injury.

Disease	Model/species	Roles and mechanism	Mobilization strategy	References
Stroke	Transient global ischemia/gerbils	Increased neurogenesis in the dentate gyrus	Mobilized by injury spontaneously	Liu et al., 1998
	dMCAOn/adult mice	Activated neural stem/progenitor cells in the pia mater	Mobilized by injury spontaneously	Nakagomi et al., 2011
	dMCAO/adult mice	Increased neurogenesis and differentiation into electrophysiologically functional neurons, astrocytes and myelin-producing oligodendrocytes in peri-infarct area	Mobilized by injury spontaneously	Nakagomi et al., 2009; Shimada et al., 2010
	Photolysis in the high vocal center/adult songbirds	Increased neurogenesis	Mobilized by injury spontaneously	Scharff et al., 2000
	MCAO/rats	Increased eNSCs proliferation and differentiation via ERK signaling pathway	Exercise	Liu et al., 2018
	MCAO/rats	Increased proliferation and differentiation of eNSCs in the bilateral hemispheres	Bilateral limb-training	Yang et al., 2012
	MCAO/juvenile rats	Increased neurogenesis and myelin repair via upregulating Wnt/β-catenin signaling pathways	Treadmill exercise	
	Bilateral common carotid arteries were occluded 5 min/Aged Gerbil	Increased neurogenesis and restoration of myelin in hippocampus	Long-term exercise	Ahn et al., 2016
	dMCAO/rats	Increased neuronal differentiation and neurogenesis in the DG of hippocampal	Enriched environment	Matsumori et al., 2006
	dMCAO/adult spontaneously hypertensive rat	Increased neural stem/progenitor cell proliferation and neurogenesis in the subventricular zone (SVZ)	Enriched environment	Komitova et al., 2005
	Bilateral common carotid artery ligation and reperfusion/mice	Increased endogenous neural stem cell proliferation via Wnt/β-catenin signaling pathway	Mallotus oblongifolius extracts	Li et al., 2020
	Photothrombotic-induced stroke/rat	Increased eNSCs proliferation and neurorestoration through Wnt/β-catenin signaling	Ellagic acid	Liu et al., 2017
	Permanent cerebral ischemia by intra-arterial injection of TiO2 spheres into MCA	Increased eNSCs proliferation and survival	Minocycline	Rueger et al., 2012
	MCAO/rats	Increased eNSCs proliferation	Skin-derived precursor cells	Mao et al., 2015
	MCAO/rats	Increased eNSCs proliferation and remyelination	NCAM-Peptide FG Loop (FGL)/NCAM mimetic peptide FGL	Klein et al., 2016
	MCAO/mice	Enhanced striatal neurogenesis	Anti-CCR2 antibody MC-21	Laterza et al., 2017
	Global brain ischemia- reperfusion/rat	Increased eNSCs proliferation and differentiation	Basic fibroblast growth factor	Ren et al., 2014
	Hypoxic-ischemic brain damage/neonatal rats	Increased eNSCs proliferation	Melatonin	Chen et al., 2019
	Hypoxic-ischemic brain damage/neonatal rats	Promotes the migration and differentiation of eNSCs	Hyperbaric oxygen	Wang et al., 2009
	Hypoxic-ischemic brain damage/neonatal rats	Increased eNSCs proliferation and differentiation	Hyperbaric oxygen	Yang et al., 2008
	Endothelin-1 induced stroke/mice	Increased eNSCs proliferation	Controlled epi-cortical delivery of epidermal growth factor	Cooke et al., 2011
	Photothrombotic stroke/adult rats	Increased neurogenesis and neuron differentiation in cortical layers II-VI and SVZ	Mobilized by injury spontaneously	Gu and Wester, 2000; Vandeputte et al., 2014
	Photothrombotic stroke/rats	eNSCs could be recruited from the cortex nearby infarct core and subventricular zone	Early expressions of hypoxia-inducible factor-1α and vascular endothelial growth factor	Song et al., 2014
	Intracerebral hemorrhage/rats	Promote the proliferation, migration and differentiation of eNSCs	Recombinant adenovirus-mediated hypoxia-inducible factor-1alpha gene	Yu et al., 2013
	MCAO/rats	Promoted eNSCs differentiation via exosomal microRNA 146b	Electro-acupuncture	Zhang et al., 2020
	MCAO/rats	Increased eNSCs proliferation and differentiation	Electro-acupuncture	Tan et al., 2018
	MCAO/rats	Increased eNSCs proliferation in the cortical peri-infarct area via the Wnt/β-catenin signaling pathway	Electro-acupuncture	Chen et al., 2015
	MCAO/rats	Promote proliferation of eNSCs and enhance angiogenesis	Transplantation of human neural stem cells via lateral ventricle injection	Ryu et al., 2016
	Hypoxic-ischemic brain damage/neonatal rats	Regulate the differentiation of eNSCs via the hedgehog signaling pathway	Transplantation of of umbilical cord blood cells via lateral ventricle injection	Zhao, 2014
	dMCAO/rats	Promote proliferation of eNSCs through vascular niches	Transplantation of Bone marrow mononuclear cells via tail vein injection	Nakano-Doi et al., 2010
	MCAO/rats	Promote eNSCs proliferation and survival	Transplantation of mesenchymal stem cells into the brain parenchyma	Yoo et al., 2008
	Intraventricular hemorrhage/rats	Increased neurogenesis and differentiated; Reduced incidence of hydrocephalus; inhibiting neuronal apoptosis.	G-CSF treatment, lithium chloride treatment, combination treatment.	Yuan et al., 2016
	Perinatal hypoxia/ischemia model/rats	Increased neural stem/progenitor cells	Mobilized by injury spontaneously	Felling et al., 2006
	Hypoxic-ischemic brain damage/mice	Increased oligodendrogenesis in SVZ	Asialo-erythropoietin	Kako et al., 2012
TBI	A needle insertion into adult rat brains/rats	Increased eNSCs proliferation	Mobilized by injury spontaneously	Wu, 1999
	TBI/rats	Increased proliferation and differentiation of eNSCs	Mobilized by injury spontaneously	Sui and Jiang, 2016
	TBI/juvenile and adult rats	eNSCs differentiated into mature neurons and integrated into the existing neuronal circuitry	Mobilized by injury spontaneously	Sun et al., 2005, 2007
	Controlled cortical impact model/mice	Increased proliferation of eNSCs	Late exercise initiation beginning at 5 weeks after trauma	Piao et al., 2013
	TBI/mice	Increased proliferation of eNSCs	Transcranial low-level laser therapy	Xuan et al., 2014
	Acute mechanical brain injury/mice	Increased proliferation of eNSCs via Notch signaling pathway	Osthole	Yan et al., 2018
	TBI/mice	Increased migration of eNSCs	EphrinB3 knockout	Dixon et al., 2016
	TBI/rats	Increased proliferation and differentiation of eNSCs	Acupuncture	Jiang et al., 2016
Neurodegenerative disease	Degenerative model of corticothalamic projection neurons/mice	Increased proliferation and differentiation of eNSCs, and formed long-distance corticothalamic connections	Mobilized by injury spontaneously	Magavi and Macklis, 2000
	Streptozotocin-induced Alzheimer/rats	Enhancing cell proliferation and suppressing apoptosis in the hippocampus via Wnt signaling pathway	Treadmill exercise	Kim et al., 2014, 2016
	6-OHDA induced Parkinson’s disease/female SD rats	Promotes the proliferation, migration and differentiation of eNSCs	Exercise	Tajiri et al., 2010
	MPTP induced Parkinson’s disease/mice	Increased proliferation of eNSCs	Endurance Exercise	Jang et al., 2018
	Alzheimer’s disease/TgCRND8 mice	Increased proliferation and differentiation of eNSCs	Enriched environment	Herring et al., 2009
Others	Huntington’s disease/rats	Increased proliferation and differentiation of eNSCs	Mobilized by injury spontaneously	Mazurová et al., 2006
	Epilepsy/rats	Enhancement of progenitor cell division in the dentate gyrus	Mobilized by injury spontaneously	Nakagawa et al., 2000
	Quinolinic acid induced Huntington’s disease/rats	Enhancing hippocampal cell proliferation	Treadmill exercise	Kim et al., 2015
	Chronically stressed rats	Restores hippocampal cell proliferation and differentiation of new born cells in the hippocampus	Enriched environment	Veena et al., 2009a,b
	Vascular dementia/rats	Increased proliferation of eNSCs via Notch signaling	Zerumbone	Sun et al., 2019
	Vascular dementia/rats	Increased proliferation of eNSCs	Ginkgo biloba extract	Wang et al., 2013
	A contusive model of spinal cord injury/rats	Increased proliferation of eNSCs and oligodendrocytes	Electroacupuncture	Wu et al., 2012
	Acute spinal cord injury/rats	Increased proliferation of eNSCs	Cetuximab modified collagen scaffold	Li et al., 2017
	Autoimmune encephalomyelitis/mice	Increased proliferation of eNSCs and myelin repair	Dibutyryl cyclic AMP	Khezri et al., 2013
	Severe combined immunodeficient/mice	Increased proliferation of eNSCs in the hippocampus	Transplantation of human MSCs in the dentate gyrus of the hippocampus	Munoz et al., 2005

eNSCs, endogenous neural stem cells; MCA, the middle cerebral artery; MCAO, middle cerebral artery occlusion; dMCAO, distal middle cerebral artery occlusion; SVZ, subventricular zone; DG, dentate gyrus; NCAM, neural cell adhesion molecule; 6-OHDA, 6-hydroxydopamine; MPTP, 1-methyl-4-phenyl-1,2,3,6-tetrahydropyridine; MSCs, human bone marrow stem cells.

## Mobilization strategies and mechanisms of endogenous neural stem cells for the repair of brain injury

### Physical exercise

Physical exercise is the therapeutic schedule commonly used for the treatment of brain injury (Liang et al., 2021). In cerebral ischemia, physical exercise was reported to enhance the proliferation and differentiation of eNSCs in the hippocampus via the ERK signaling pathway in a rat model of cerebral infarction (Liu et al., 2018). In a Wistar rat model induced by middle cerebral artery occlusion, bilateral limb-training was observed to improve the proliferation and differentiation of eNSCs in the bilateral hemispheres and accelerate the recovery of neurologic function (Yang et al., 2012). The findings from the group of Ji Hyeon Ahn showed that long-term exercise improved memory impairment through enhanced neurogenesis, restoration of myelin and microvessel damage, and decreased synaptic adhesion molecule induced by a stroke in the aged gerbil hippocampus (Ahn et al., 2016, 2018). The undergoing mechanism of neurogenesis and myelin repair involved activated Wnt/β-catenin signaling pathways and upregulated BDNF (Alcantara et al., 2018; Cheng et al., 2020). In the injury of TBI, physical exercise enhanced the generation of new neurons by activating the eNSCs in the hippocampus and promoted cognitive recovery (Piao et al., 2013). Similarly, physical exercise ameliorated the learning and memory impairment induced by Alzheimer’s disease through increasing cell proliferation and suppressing apoptosis in the DG (Azimi et al., 2018; Jahangiri and Hosseini, 2019). In Parkinson’s Disease, physical exercise induced neurogenesis and increased the TH-immunopositive neurons in SN, and attenuated the loss of dopaminergic neurons (Tajiri et al., 2010; Petzinger et al., 2013; Jang et al., 2018; Palasz et al., 2019). Using streptozotocin-induced Alzheimer model, Kim et al. (2014, 2016) found that postnatal treadmill exercise enhanced cell proliferation and suppressed apoptosis in the hippocampus through Wnt signaling pathway. In addition, physical therapy, such as low-level laser therapy and deep brain stimulation (DBS), provided the neuroprotective effects through enhancing neurogenesis in brain injury (Xuan et al., 2014; Tsai et al., 2019).

### Enriching environments

Enriching environments (EE) that consists of physical, social and sensory stimuli are effective treatment measures for the normal and injured brain through promoting neurogenesis and plasticity (Hannan, 2014). There is plenty of evidence that EE improved spatial learning ability in neurological disease (Olson et al., 2006; Gelfo et al., 2011; Kempermann, 2019). EE induces various molecular and cellular changes in the hippocampus, including upregulated the expression of neurotrophic factors, enhanced neurogenesis, and synaptic plasticity (Pham et al., 2002; Bekinschtein et al., 2011). The disturbed neurogenesis induced by cerebral ischemia has been restored by EE, and the density of NeuN positive cells has been enhanced (Matsumori et al., 2006). In rats with stroke, EE promoted neural stem/progenitor cell proliferation in the SVZ and ameliorated the impairment in spatial learning and memory, and passive avoidance memory and long-term potentiation (LTP) (Komitova et al., 2005; Ahmadalipour et al., 2017; Wang et al., 2019). EE increased the number of newborn mature hippocampal neurons and upregulated the expression of multiple plasticity associated molecules compared to the standard housed control mice in transgenic mice with Alzheimer-like pathology (Herring et al., 2009). The results from David and Lisa group respectively showed that EE improved cognitive impairment in AD transgenic mice through a dual mechanism in both amyloid dependence and independence (Costa et al., 2007; Robison et al., 2020). Exposure to an enriched environment ameliorated depressive symptoms by promoting the survival and differentiation of eNSCs in the hippocampus in chronically stressed rats (Veena et al., 2009a,b). Despite EE has been demonstrated the efficacy in the treatment of brain injury through enhancing neurogenesis and plasticity, there was still a long way to apply to the clinical setting because of the lack of standards (McDonald et al., 2018).

### Small molecule drugs and herbal extract

There are many exogenous small molecule drugs reported to be capable of triggering the proliferation of eNSCs (Wu et al., 2017). Zerumbone is a sesquiterpenoid and cyclic ketone, which is obtained by steam distillation from Zingiber zerumbet Smith, a type of edible ginger found in southeast Asia particularly (Pubchem, 2022). 50 mg/kg and 100 mg/kg intraperitoneally Zerumbone was reported to stimulate the proliferation of neural stem cells in rats by regulating the Notch signaling (Sun et al., 2019). Osthole was found to promote eNSC proliferation and improve neurological function via the Notch signaling pathway in mice with acute mechanical brain injury (Yan et al., 2018). In a mouse model of ischemic injuries induced by bilateral common carotid artery ligation, mallotus oblongifolius was identified to improve the morphology of neurons, resist the loss of neurons, and enhance the content of the nestin protein in the cerebral cortex and subgranular zone area (Li et al., 2020). *In vitro*, mallotus oblongifolius was observed to activate the Wnt/β-catenin signaling pathway, increase the proliferation of neural stem cells, and enhance the protein expression levels of β-catenin and CyclinD1 in the oxygen-glucose deprivation and reperfusion (OGD/R) treated cell model. EGb761, one of the ginkgo biloba extracts, enhanced the proliferation of neural stem cells in the subventricular zone and DG in a rat model of vascular dementia (Wang and Wang, 2013). In a photothrombotic-induced rate model of brain injury, administration of polyphenol ellagic acid could improve brain injury outcomes and increase the proliferation of neural stem cells through the Wnt/β-catenin signaling pathway (Liu et al., 2017). Cetuximab is an EGFR signaling antagonist, which was found to significantly promote neurogenesis in the lesion site of a rat model of severe spinal cord injury (SCI). On the other hand, it was also indicated that implanted cetuximab modified linear ordered collagen scaffolds (LOCS) into SCI lesion sites in dogs leading to neuronal regeneration including neuronal differentiation, maturation, myelination, and synapse formation (Li et al., 2017). Aromatic-turmerone derived from Curcuma longa, which is an herb of the Zingiberaceae family, was reported to promote the mobilization of endogenous stem cells resident in the nervous system (Androutsellis-Theotokis, 2014). Minocycline was observed to increase eNSCs in both the SVZ as well as the hippocampus in a rat stroke model. In addition, minocycline showed positive effects on eNSC survival (Rueger et al., 2012). Hyperbaric oxygen (HBO) showed the capacity to improve the proliferation of neural stem cells in the SVZ and DG, promote the eNSCs to migrate to the cortex and differentiate into mature neurocytes in neonatal rats with hypoxic-ischemic brain damage (HIBD) (Yang et al., 2008; Wang et al., 2009), which was correlated with the activation of Wnt signaling (Wang et al., 2007).

### Cytokines

Some cytokines are also reported to promote the proliferation of eNSCs. The repressor element-1 silencing transcription factor (REST), polycomb group (PcG) proteins and other chromatin remodeling factors play important roles in neural stem and progenitor cell biology, and can be applied to potentially promote eNSCs- mediated brain repair (Mehler, 2011). The anti-CCR2 antibody MC-21 was used to deplete circulating monocytes during the first week after stroke, which enhanced striatal neurogenesis 1 week later, probably by means of improving the short-term survival of the newly formed neuroblasts in the subventricular zone and close to the striatum in a mouse model (Laterza et al., 2017). After subcutaneously injected on cellular compartments affected by degeneration and regeneration after stroke, the FG loop (FGL) peptide profoundly promoted the mobilization of eNSCs in the neurogenic niches and triggered remyelination (Klein et al., 2016). The chitosan-neurotrophic factor 3 was applied to bridge the transected spinal cord and release neurotrophic factor 3 slowly to provide a favorable microenvironment for neural stem cell activation and migration (De Filippis and Pang, 2015). It was indicated that the slow release of neurotrophic factor 3 by chitosan promoted physiological repair processes in SCI in an animal model, implying NT3 contributed to the generation of neurons. Skin-derived precursor cells could secrete basic fibroblast growth factor (bFGF) and vascular endothelial growth factor (VEGF) in the ischemic region and further significantly promote the proliferation of endogenous nestin (+) and βIII-tubulin (+) neural stem cells in cerebral ischemia rats (Mao et al., 2015). Increased expression of epidermal growth factor (EGF) and bFGF after human cerebral infarction probably led to endogenous reparation and it correlated with the proliferation of eNSCs in humans (Duan et al., 2008). In a rat model of the cerebral cortex with global brain ischemia-reperfusion, basic fibroblast growth factor (bFGF) could be used to promote and extend the proliferation of eNSCs *in situ*, as well as to promote the differentiation of eNSCs into neurons (Ren et al., 2014). Controlled epi-cortical delivery of EGF was used to stimulate eNSCs proliferation in a mouse model of stroke (Cooke et al., 2011). The non-erythropoietic derivative (asialo-erythropoietin) had been confirmed to promote the maturation of SVZ-derived oligodendrocyte progenitor cells and improve neurological function in a Hypoxic-ischemic brain damage mice (Kako et al., 2012). In the rats with hypoxic-ischemic brain damage, single-dose immediate melatonin treatment and 7-day continuous melatonin treatment could improved the proliferation of eNSCs and ameliorated long-term histological injury in the brain of neonatal (Chen et al., 2019). In fetal Sprague-Dawley rats, neural stem cells derived from hippocampi were treated with ciliary neurotrophic factor for 7 days, which showed an increased number of microtubule associated protein-2-positive cells and decreased number of glial fibrillary acidic protein-positive cells (Zhang et al., 2016). The administration of intraventricular growth factors promoted eNSCs mobilization (Nakatomi and Saito, 2016). Using an ephrinB3 knockout mice model, Dixon et al. (2016) found that ephrinB3 expression in tissues surrounding neurogenic regions functioned to restrict neuroblast migration outside the rostral migratory stream (RMS) by limiting chain migration. The early expressions of hypoxia-inducible factor-1α (HIF-1α) and VEGF were observed to promote the proliferation, migration and differentiation of eNSCs in a rat stroke and ICH model (Yu et al., 2013; Song et al., 2014). Dibutyryl cyclic AMP (dbcAMP) might serve as a potential treatment option for inducing myelin repair in the context of demyelinating diseases like multiple sclerosis partially because of the eNSCs and their increased recruitment (Khezri et al., 2013).

### Acupuncture and moxibustion

In addition, electro-acupuncture was identified to promote the differentiation of eNSCs. Electro-acupuncture was reported to promote the differentiation of eNSCs via exosomal microRNA 146b after ischemic stroke in rats (Zhang et al., 2020). Electro-acupuncture also promoted the proliferation and differentiation of eNSCs probably via modulating PRG5/RhoA signaling (Tan et al., 2018). In a rat model of SCI at spinal segment T8-9, the electroacupuncture of acupoints Huantiao (GB30) and Huatuojiaji (Ex-B05) significantly increased the numbers of BrdU(+)/NG2(+) cells at spinal cord tissue 15 mm away from the injury center in the rostral and caudal directions (Wu et al., 2012). Acupuncture was reported to induce the proliferation and differentiation of eNSCs, therefore neural repair was promoted in traumatic brain injury rats (Jiang et al., 2016). In a raumatic brain model established by Feeney’s free fall epidural impact method, the acupuncture at acupoints (Baihui, Shuigou, Fengfu, Yamen, and bilateral Hegu) increased significantly the number of nestin-expressing cells (Nestin^+^), bromodeoxyuridine/glial fibrillary acidic protein (BrdU^+^/GFAP^+^), BrdU/S100 calcium-binding protein B, BrdU/microtubule-associated protein 2- and BrdU/galactocerebrosidase-positive cells (Jiang et al., 2016). The results from Chen Bin et al. indicated that electro-acupuncture significantly improved neurological deficits, reduced the infarct volume and enhanced NPC proliferation through the Wnt β-catenin signaling pathway (Chen et al., 2015).

### Transplantation of exogenous cells

Transplantation of exogenous cells was found to improve the proliferation of eNSCs. After transplanted into the subventricular zone, human neural stem cells in the brain of a rat model of focal cerebral ischemia triggered the proliferation of eNSCs and their differentiation into mature neural-like cells, and improved angiogenesis (Ryu et al., 2016). Umbilical cord blood mononuclear cells (UCBMC) were reported to probably promote eNSCs proliferation, ameliorate brain injury, and reduce glial differentiation in hypoxia-ischemia neonatal rats through the Sonic hedgehog (Shh) signaling pathway (Zhao, 2014). Administration of bone marrow mononuclear cells (BMMCs) could contribute to the proliferation of endogenous ischemia-induced neural stem/progenitor cells through vascular niche regulation in a mouse model of cortical infarction, which includes regulation of endothelial proliferation (Nakano-Doi et al., 2010). Bone marrow-derived mesenchymal stem cells (MSCs) were transplanted into the brain parenchyma 3 days after induction of stroke by occluding the middle cerebral artery for 2 h, which promoted the proliferation of eNSCs and suppressed the cell death of newly generated cells. The newborn cells were observed to migrate toward ischemic territory and differentiate in ischemic boundaries into doublecortin + neuroblasts at higher rates in animals with mesenchymal stem cells (MSCs) compared to the control group, partly because MSCs had properties of enhancing endogenous neurogenesis and protecting newborn cells from the deleterious environment (Yoo et al., 2008). The implanted human MSCs significantly promoted the proliferation of eNSCs which expressed the stem cell marker Sox2 (Munoz et al., 2005) (see Table 1 in detail).

### Negative factors in endogenous neural stem cells mobilization

There are also some factors reported to curb the proliferation of eNSCs. Gli1 is a gene that encodes a member of the Kruppel family of zinc finger proteins. The encoded transcription factor is activated by the sonic hedgehog signal transduction cascade and regulates stem cell proliferation (NCBI, 2021). eNSCs can be mobilized for the repair of demyelinated lesions by inhibiting Gli1 (Samanta et al., 2015). Methylprednisolone was found to inhibit the proliferation of eNSCs in cynomolgus monkeys with SCI (Ye et al., 2018). Chronic alcohol consumption have a negative impact on the survival of neural stem cells in the subventricular zone and altered neural stem cell differentiation in the subventricular zone, subgranular zone, and tanycyte layer in an inducible transgenic mouse model (McGrath et al., 2017). The cytosine arabinoside inhibited the activation of eNSCs (Zhang et al., 2016). eNSCs were observed the proliferation and differentiation in a mild SCI in rats, while the ability to differentiate into neurons is limited partly because of the high expression of inhibitory Notch1 and Hes1 genes after injury (Liu et al., 2015).

## Summary and outlook for endogenous neural stem cells in the repair of brain injury

Neural stem cells have proven strikingly valuable to the repair in brain injury. Although exogenous neural stem cells are a potential strategy for repairing brain injuries, it is not popular because of tumorigenic, immunological, and ethical problems. As a result, eNSCs appear to be more practical in brain repair. However, there are still lots of unsolved difficulties which limit the maximum therapeutic effect of eNSCs. Firstly, although the brain injury and some exogenous medicines can trigger neurogenesis in SVZ and hippocampus DG, even some of them can migrate to the site of ischemic area, most of them die during the next few weeks after stroke. Thus we need to explore some strategies to enhance the number of surviving neurons, increased cell survival rate. Secondly, in addition to the role of nutrient factor secretion, the differentiation of eNSCs into mature neurons is more important for repair of brain injury. The new-formed neurons can replace the injured neurons and repair the disrupted neural network. However, at present the mechanisms that affected differentiation of eNSCs is not understood clearly and the effective promotion strategies is still lack. Finally, a single treatment strategy may not be enough to maximize the effect of eNSCs. Some strategies are better at promoting eNSCs proliferation, while others are better at promoting eNSCs migration or differentiation. The comprehensive treatment program that integrated small drugs, neurotrophic factor and growth factor, rehabilitation, electro-acupuncture, and so on may lead to a better outcomes. Thus we need to further investigate the optimal combination of treatment options and the optimum application time window of every option.

## Author contributions

PZ and KY developed the idea and revised the manuscript. QH, WL, YY, YJ, and DW reviewed the literature and prepared the tables. HL and TW prepared and revised the manuscript. All authors have read and approved the final manuscript.
